# Molecular Specialization of GABAergic Synapses on the Soma and Axon in Cortical and Hippocampal Circuit Function and Dysfunction

**DOI:** 10.3389/fnmol.2019.00154

**Published:** 2019-06-26

**Authors:** April Contreras, Dustin J. Hines, Rochelle M. Hines

**Affiliations:** Department of Psychology, University of Nevada, Las Vegas, Las Vegas, NV, United States

**Keywords:** GABAergic synapse development, epilepsies and epileptic syndromes, cholecystokinin, parvalbumin, interneuron, GABA_A_ receptor subunits, somatic inhibitory synapse, axon initial segment inhibitory synapse

## Abstract

The diversity of inhibitory interneurons allows for the coordination and modulation of excitatory principal cell firing. Interneurons that release GABA (γ-aminobutyric acid) onto the soma and axon exert powerful control by virtue of proximity to the site of action potential generation at the axon initial segment (AIS). Here, we review and examine the cellular and molecular regulation of soma and axon targeting GABAergic synapses in the cortex and hippocampus. We also describe their role in controlling network activity in normal and pathological states. Recent studies have demonstrated a specific role for postsynaptic dystroglycan in the formation and maintenance of cholecystokinin positive basket cell terminals contacting the soma, and postsynaptic collybistin in parvalbumin positive chandelier cell contacts onto the AIS. Unique presynaptic molecular contributors, *LGI2* and *FGF13*, expressed in parvalbumin positive basket cells and chandelier cells, respectively, have also recently been identified. Mutations in the genes encoding proteins critical for somatic and AIS inhibitory synapses have been associated with human disorders of the nervous system. Dystroglycan dysfunction in some congenital muscular dystrophies is associated with developmental brain malformations, intellectual disability, and rare epilepsy. Collybistin dysfunction has been linked to hyperekplexia, epilepsy, intellectual disability, and developmental disorders. Both *LGI2* and *FGF13* mutations are implicated in syndromes with epilepsy as a component. Advancing our understanding of the powerful roles of somatic and axonic GABAergic contacts in controlling activity patterns in the cortex and hippocampus will provide insight into the pathogenesis of epilepsy and other nervous system disorders.

## Introduction

**FIGURE 1:**
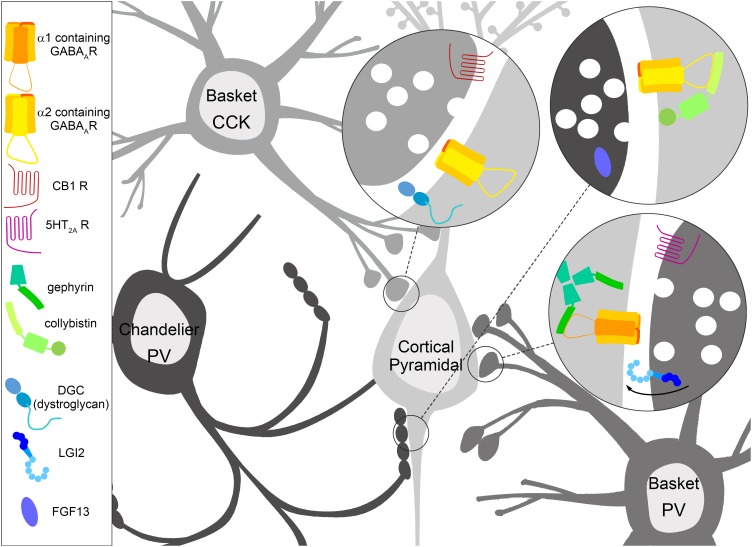
Specialization of inhibitory GABAergic synapse subtypes. Interneurons have molecular specifications which help guide, form, and maintain GABAergic synapses onto distinct areas of the cortical pyramidal cell, which in turn feature molecular specialization in terms of enriched GABA_A_ receptor (GABA_A_R) subtypes and interacting proteins. Cholecystokinin (CCK) positive basket cells target the soma of pyramidal cells, where the dystrophin glycoprotein complex (DGC) containing dystroglycan and GABA_A_Rs containing the α2 subunit are robustly expressed. The CCK positive presynaptic terminal is enriched with Cannabinoid receptor type 1 (CB1). Parvalbumin (PV) positive basket cells target the soma of pyramidal cells enriched with GABA_A_Rs containing the α1 subunit anchored by gephyrin. The PV positive presynaptic terminal contains the serotonin 5-HT_2A_ receptor, which is thought to depolarize PV positive basket cells. LGI2 protein is enriched in PV positive basket cells during synaptogenesis, and regulates the formation of these synapses. PV positive chandelier cell cartridges target the axon initial segment (AIS) of pyramidal cells. GABA_A_Rs containing the α2 subunit are enriched here, and collybistin interaction plays a key role in AIS localization, although both collybistin and α2 are found at other inhibitory contact sites. The non-secreted protein FGF13 is enriched in PV positive chandelier cells during synaptogenesis, and regulates the formation and maintenance of these synapses.

The functional output of the nervous system relies upon coordinated patterns of activity within neuronal circuitry. Neuronal circuits in the cortex and hippocampus are composed of not only excitatory pyramidal cells, but a multitude of diverse interneuron types that express unique complements of proteins and play distinct functional roles (Petilla Interneuron Nomenclature Group et al., 2008). The diversity of inhibitory interneuron signaling allows for multiple levels of modulation of excitatory principal cell firing (Kubota et al., 2016). Interneurons release the neurotransmitter γ-aminobutyric acid (GABA) onto postsynaptic targets, which then binds to GABA_A_ receptors (GABA_A_Rs). Diversity is also present in the postsynaptic targets of interneuron types (Figure 1). In particular, interneurons releasing GABA onto the principal cell soma and axon exert powerful control by virtue of proximity to the site of action potential generation at the axon initial segment (AIS; Miles et al., 1996; Klausberger and Somogyi, 2008). As with specialization of the presynaptic interneuron partner, the postsynapse is also specialized by enrichment of GABA_A_R subtypes. GABA_A_Rs are heteropentamers, and those enriched at the postsynapse are most commonly composed of 2α, 2β, and a γ subunit. Some of the postsynaptic specialization of GABA_A_Rs is conferred by the α subunit, with α1 containing receptors enriched on the dendrites and soma, and α2 containing receptors enriched on the soma and AIS (Jacob et al., 2008). Further complexity is added due to brain circuits likely relying on unique mechanisms to control synapse targeting, specificity, and molecular specialization. In this review, we examine the cellular and molecular regulation of soma and axon targeting GABAergic synapses in the cortex and hippocampus, as well as clarify their role in controlling network activity in these respective circuits. Because unique mechanisms likely exist in each circuit, we will compare and contrast soma and axon targeting GABAergic synapses in the cortex and hippocampus based on current research. We also examine the role of soma and axon targeting GABAergic synapse dysfunction in pathological states, linking animal phenotypes and human syndromes to key molecular contributors at soma and axon targeting synapses of cortical and hippocampal circuits.

## Soma and Axon Targeting Interneurons

The function of a neuronal circuit relies upon inhibitory interneuron modulation of principal cell activity, with interneurons that contact the perisomatic region exerting powerful control over axonal output of principal cells (Miles et al., 1996). The cortex and hippocampus feature complex circuitry exemplified by interneuron diversity. Interneurons can be classified by their morphology, connectivity, firing pattern, and gene expression pattern. Based on morphology, 16 or more types of interneuron have been distinguished in the hippocampus (Cajal, 1893; Lorente and De Nó, 1934; Parra et al., 1998) while transcriptomic cell typing has identified 23 interneuron types in the cortex (Tasic et al., 2016). Interneurons targeting the perisomatic region generally have a small number of large terminals in comparison to those contacting dendrites. While dendrite-targeting inhibitory synapses can suppress Ca^2+^ dependent spiking, those contacting the perisomatic region can suppress repetitive discharge of Na^+^ dependent action potentials (Miles et al., 1996). Despite the morphological and molecular complexity of cortical and hippocampal circuits, the majority of interneurons in these regions express either the neuropeptide cholecystokinin or the calcium binding protein parvalbumin (CCK; PV; Whissell et al., 2015).

CCK positive basket cells target the soma and proximal dendrites of cortical and hippocampal pyramidal cells (Figure 1), and do not appear to innervate the AIS (Panzanelli et al., 2011). Proportions of CCK basket cells express the ionotropic serotonin receptor (5-HT_3_; Morales and Bloom, 1997) and the metabotropic Cannabinoid receptor type 1 (CB1), which modulate GABA release from the presynaptic terminal (Katona et al., 1999; Lee and Soltesz, 2011). Functionally, CCK positive basket cells provide long-lasting inhibition, modulating cortical and hippocampal cell activity based upon motivation, emotion, and autonomic information from subcortical regions (Buzsáki, 1996; Freund and Katona, 2007).

PV positive interneurons include basket cells which target the soma and proximal dendrites of excitatory pyramidal cells, and chandelier cells whose terminals synapse onto the AIS (Figure 1; Defelipe et al., 1985). PV positive basket cell terminals express the metabotropic serotonin receptor 5-HT_2A_, with electrophysiological data showing that activation of 5-HT_2A_ depolarizes PV positive GABAergic interneurons (Weber and Andrade, 2010). PV positive basket and chandelier cells are fast-spiking, with the potential to robustly influence the activity of hundreds of pyramidal cells (Hu et al., 2014), and are responsible for the generation of network oscillations in both the cortex and hippocampus (Freund and Buzsáki, 1996). While both CCK positive and PV positive interneurons inhibit the perisomatic region of pyramidal cells, they feature molecular specialization and have unique functional contributions to network activity.

## Molecular Specialization of Postsynaptic Sites on the Soma and Axon

Since the identification of distinct interneuron types, research has focused on the unique molecular and functional characteristics of their synaptic specializations. CCK positive somatic terminals are enriched with GABA_A_Rs containing the α2/α3 subunits (Nyíri et al., 2001), and the formation and maintenance of CCK terminals is linked to dystroglycan (DG) of the dystrophin glycoprotein complex (DGC; Figure 1; Früh et al., 2016). The DGC is composed of a number of interacting proteins dependent upon tissue type, with brain DGC including dystrophin (or utrophin), syntrophin, dystrobrevin, and DG. Pyramidal cells express DGC in perisomatic clusters postsynaptic to GABAergic terminals (Knuesel et al., 1999; Brünig et al., 2002; Lévi et al., 2002). DG interacts with neurexins to form GABAergic synapses (Sugita et al., 2001), and disruption of the DGC alters synaptic clustering of GABA_A_Rs (Knuesel et al., 1999; Vaillend et al., 2010).

Conditional deletion of DG (DG cKO) leads to a loss of the DGC and a modification in GABA_A_R subunit clustering, but does not prevent the formation of GABAergic terminals (Table 1; Früh et al., 2016). DG cKO mice exhibit a decrease in cluster size of GABA_A_Rs containing the α1 subunit, along with an increase in cluster density of GABA_A_Rs containing the α2 subunit. Examination of DG cKO mice reveals a specific loss of CCK positive basket cell terminals onto pyramidal cells of the cortex and hippocampal CA1. Induction of DG cKO in mature mice reveals that DG is necessary for the maintenance of CCK positive basket terminals, with the absence of DG leading to a reduction in already formed connections with CCK positive terminals (Früh et al., 2016). The role of DG in the maintenance of CCK positive terminals is independent of neurexin, demonstrated using mice that express the T190M variant of DG, which lacks neurexin binding. Disruption of DG alters functional connectivity of CCK positive terminals, with administration of carbachol to DG cKO slices showing a loss of carbachol-induced increases in inhibitory currents (Früh et al., 2016). Carbachol increases perisomatic inhibitory transmission in pyramidal cells in control slices (Früh et al., 2016), which is mediated by direct excitation of CB1 receptor expressing CCK positive interneurons (Nagode et al., 2014). DG cKO mice also appear to have a reduction in body and brain weight compared to controls (Früh et al., 2016).

**Table 1:** Overview of key proteins involved in specification and maintenance of soma and axon targeting inhibitory synapses, and implications for disorders of the nervous system.

Mouse model	Molecular/cellular phenotype	Network/behavioral phenotype	Associated disorders
*Gabra2*-1 (Hines et al., 2018)	↓ α2 subunit interaction with collybistin *in vitro* ↑ total α2 in hippocampus, cortex ↓ total collybistin in hippocampus, cortex ↓ α2 containing receptors at cortical cell AIS ↓VGAT positive terminals onto cortical cell AIS	Gabra2-1 mouse model (Hines et al., 2018): Reduced amplitude and decay of sIPSC in hippocampal CA1; Spontaneous seizures during development; Developmental mortality (peaks ∼ PND 20); Elevations in δ-power in surviving adults; Increased anxiety in light-dark boxes and elevated plus maze	α2 subunit - Generalized epilepsy associated with *GABRA2* (The International League Against Epilepsy Consortium on Complex Epilepsies, 2018) Collybistin - Hyperekplexia (Striano and Zara, 2017) - Epilepsy (Wang et al., 2018) - Anxiety and aggression (Kalscheuer et al., 2009) - Mental retardation (Shimojima et al., 2011)
Nex-Cre/*Dag1* conditional KO (DG cKO) (Früh et al., 2016)	↓ perisomatic dystrophin-glycoprotein complex (DGC) in hippocampus ↓ α1 subunit cluster size ↑ α2 subunit density ↓ CCK basket cell terminals onto hippocampal, cortical cell soma	DG cKO (Früh et al., 2016): Carbachol induced increase in inhibitory currents in slice; No change in hippocampal CA1 sIPSC frequency or amplitude; Reduced body and brain weight; Peak mortality at 10 weeks	Dystrophin - Duchenne muscular dystrophy (DMD; McNally and Pytel, 2007) Dystroglycan - DMD associated with mental retardation (Knuesel et al., 1999; Daoud et al., 2009; Desguerre et al., 2009)
Lhx6^Cre/+^*shLgi2* (Favuzzi et al., 2019)	↓ density of presynaptic inputs onto cortical cell soma	Canine Benign Familial Juvenile Epilepsy (Seppälä et al., 2011): Unilateral epileptic discharges in central-parietal and occipital lobes; Epilepsy onset at 5–9 weeks with remission by 4 months; Seizures and whole body tremors	- Epilepsy Canine Benign Familial Juvenile Epilepsy; (Seppälä et al., 2011); Partial Epilepsy with Pericentral Spikes (Kinton et al., 2002; Limviphuvadh et al., 2010)
Nkx2-1^CreER/+^*shFgf13* (Favuzzi et al., 2019)	↓ density of presynaptic inputs onto cortical cell AIS Axonal disorganization in *Fgf13* deficient PV positive chandelier cells when downregulated at P2	Fgf13 +/- (Puranam et al., 2015): Frequency of IPSCs amplitude of mIPSCs reduced in whole cell recordings in hippocampal slice; Age-dependent susceptibility to hyperthermia-induced seizures ≤ PND15 (Puranam et al., 2015); *Fgf13* mutation lethal in male offspring Spontaneous recurrent seizures	- Epilepsy (Guillemot and Zimmer, 2011); Febrile Seizures Plus (Puranam et al., 2015); Borjeson-Forssman-Lehmann syndrome (Malmgren et al., 1993; Gecz et al., 1999)

PV positive cells terminate onto the soma and AIS, with synapses on the AIS enriched with GABA_A_Rs containing the α2 subunit (Figure 1; Nusser et al., 1996; Nyíri et al., 2001). Recently, the α2 subunit was shown to have a strong interaction with the collybistin-SH3 domain, but a relatively weak interaction with the gephyrin-E domain (Hines et al., 2018). Conversely, the α1 subunit interaction with the collybistin-SH3 domain is relatively weak, with the gephyrin-E domain interaction being strong. These studies also showed that *in vitro*, collybistin (CB) and gephyrin compete for interaction with the α2 subunit (Hines et al., 2018). This sets interaction with CB as a possible means of regulating postsynaptic enrichment of α2 subunit containing receptors. To examine this possibility, a substitution mutation was made to introduce the gephyrin-preferring portion of the α1 subunit large intracellular loop into α2 (*Gabra2*-1). The *Gabra2*-1 mutation reduces interaction with CB, and results in an increase in total α2, but a decrease in CB expression in both the cortex and hippocampal CA1 (Table 1; Hines et al., 2018). The *Gabra2*-1 mutation reduces clustering of α2-containing receptors, but does not appear to reduce the overall size or density of inhibitory presynaptic terminals stained by VGAT or GAD65 (Hines et al., 2018). Specific examination of AIS synapses showed a loss of α2-containing receptors, and a loss of VGAT positive terminals opposed to the AIS (Hines et al., 2018). A subset of heterozygous and homozygous *Gabra*2-1 pups die during postnatal (PN) development, with a peak in mortality at postnatal day 20, and during this time spontaneous seizures are observed. *Gabra2*-1 mice also show abnormalities in electroencephalogram (EEG) recordings, with elevations in δ-power (Hines et al., 2018).

## Molecular Specialization of Soma and Axon Targeting Interneurons

During synaptogenesis, contact between opposite yet complementary pre- and post-synaptic terminals is essential for proper circuit formation. In addition to postsynaptic specializations on pyramidal cells, recent papers have identified molecular specialization of presynaptic interneuron subtypes critical for their postsynaptic targeting. Cell sorting of interneurons during peak synaptogenesis, followed by RNA-sequencing and whole-transcriptome analyses, has recently identified molecular programs for synaptogenesis specific to soma, axon, and dendrite targeting interneurons (Favuzzi et al., 2019). Gene ontology analysis showed that the most enriched genes are those belonging to synaptic membrane compartments and processes that contribute to synaptogenesis, which were not enriched in mature cortex (Favuzzi et al., 2019). *Lgi2*, a member of the leucine-rich glioma inactivated protein gene family, was identified as a chief regulator for the establishment of perisomatic inhibitory synapses by a population of PV positive basket cells (Figure 1; Favuzzi et al., 2019). *Lgi2* encodes a secreted protein (LGI2) that consists of leucine-rich repeat and epilepsy-associated/epitempin (EPTP) domains. Prior studies have implicated the related family member LGI1 in maturation of excitatory synapses (Senechal et al., 2005; Kegel et al., 2013). LGI proteins have been shown to interact with a disintegrin and metalloprotease (ADAM) proteins (Seppälä et al., 2011).

Through cell sorting *Fgf13* was identified as a candidate for AIS-targeting chandelier synapses (Figure 1; Favuzzi et al., 2019). *Fgf13* is a member of the fibroblast growth factor gene family, which encodes proteins (FGFs) critical for development (Wu et al., 2012; Pablo et al., 2016). Unlike many FGF family members, FGF13 is non-secretory and functions independent of FGF receptors. FGF13 has been shown to be a microtubule stabilizing protein enriched in the growth cones of cortical cells (Wu et al., 2012). FGF13 is also known to limit localization of voltage-gated sodium channels to the somatodendritic compartment of principal neurons, while FGF14 promotes localization to the proximal axon (Pablo et al., 2016).

To investigate the role of *Lgi2* and *Fgf13*, interneuron cell type-specific Cre-driver lines were combined with adeno-associated virus (AAV) vectors carrying miR-based short-hairpin RNAs (Favuzzi et al., 2019). Cell-specific down-regulation of *Lgi2* and *Fgf13* led to a decrease in density of presynaptic inputs from interneurons expressing the short-hairpin RNAs. A decrease in somatic inhibitory synapses made by PV positive basket cells was observed upon down-regulation of *Lgi2* at P2 (Table 1; Favuzzi et al., 2019). ADAM22, the proposed postsynaptic partner of expressed LGI2 was also shown to be colocalized with gephyrin clusters on the soma, opposite GAD-65+ terminals (Favuzzi et al., 2019). Interestingly, *Fgf13* deficient PV positive chandelier cells showed axonal disorganization in addition to a loss of AIS innervation when down regulation was induced at P2 (Table 1; Favuzzi et al., 2019). Axonal disorganization itself may contribute to the decrease in AIS innervation observed, although this may also represent a dual role for FGF13. Interestingly, chandelier cell synaptic boutons were decreased in the absence of axonal disorganization if *Fgf13* was down-regulated after P14, confirming that expressed FGF13 also plays a role in maintenance of chandelier cell contacts onto the AIS, after the axon has reached its target (Favuzzi et al., 2019).

## Implications of Gabaergic Synapses on the Soma and Axon in Disorders of the Nervous System

The coordination of excitatory principal cell firing relies on interneuron function, and dysregulation of soma and axon targeting interneurons has been identified in disorders of the nervous system (DeFelipe et al., 1993; Rubenstein and Merzenich, 2003; Ali Rodriguez et al., 2018). Disruption of neuronal DG directly impacts the maintenance of CCK positive basket terminals onto pyramidal cell somas, leading to impaired CCK positive interneuron mediated neurotransmission and functional connectivity. Mutations in DGC components such as dystrophin are the most common cause of muscular dystrophies, which are movement disorders characterized by a robust degeneration of muscle tissue (Table 1; McNally and Pytel, 2007). Muscular dystrophies with neurological aberrations can be caused by varying genetic mutations, and are associated with a lack of available glycosylated DG (Table 1; Brancaccio, 2005; Barresi and Campbell, 2006). Varying ranges of intellectual disability have been identified in individuals with muscular dystrophies, and cognitive deficits are associated with neuronal DG alterations (Knuesel et al., 1999; Moore et al., 2002; Daoud et al., 2009; Desguerre et al., 2009; Vaillend et al., 2010).

Disruption in inhibitory signaling mediated by PV positive cells on a global level has been associated with neurodevelopmental disorders (Ali Rodriguez et al., 2018). Schizophrenia, through post-mortem studies and in animal models, has been associated with soma and axon targeting inhibitory synapses (Lewis et al., 2008, 2012; Hines et al., 2013). Autism spectrum and related disorders such as Angelman syndrome and Rett syndrome have also been linked to PV cell dysfunction, and notably these disorders have a high incidence of epilepsy (Table 1; Kalscheuer et al., 2009; Shimojima et al., 2011; Ali Rodriguez et al., 2018). Mutations in the GABA_A_R subunit genes have been implicated in genetic epilepsies (Baulac et al., 2001; Wallace et al., 2001; Hines et al., 2018). The gene encoding the α2 subunit (*GABRA2*) was identified as one of the most likely biological epilepsy genes in a recent genome-wide mega-analysis (The International League Against Epilepsy Consortium on Complex Epilepsies, 2018). In the *Gabra2*-1 animal model, altered clustering of GABA_A_Rs containing the α2 subunit led to developmental seizure and mortality, as well as anxiety-like phenotypes (Hines et al., 2018). In humans, mutations in the gene encoding collybistin (*ARHGEF9*), lead to hyperekplexia syndromes that include intellectual disability and mental retardation (Table 1; Shimojima et al., 2011; Striano and Zara, 2017). *ARHGEF9* mutations are also associated with epilepsies and anxiety in humans (Kalscheuer et al., 2009; Wang et al., 2018).

*LGI2* and *FGF13* dysfunction have also been linked to epilepsy. *LGI1* mutations account for about half of Autosomal Dominant Lateral Temporal lobe Epilepsy (ADLTE; Kalachikov et al., 2002). Mutations in *LGI2* have also been associated with an epileptic phenotype, especially that of canine Benign Familial Juvenile Epilepsy (Table 1; Fukata et al., 2010; Seppälä et al., 2011). LGI2 is also a leading candidate for mutations in the 4p15 region thought to be responsible for Partial Epilepsy with Pericentral Spikes (PEPS; Kinton et al., 2002; Limviphuvadh et al., 2010). Mutations in *FGF13* are linked to Genetic Epilepsy and Febrile Seizures Plus (GEFS+; Guillemot and Zimmer, 2011; Puranam et al., 2015), as well as Börjeson-Forssman-Lehmann syndrome, which is a rare X-linked disorder characterized by intellectual disability, obesity, seizures, hypogonadism, and distinctive facial features (Table 1; Malmgren et al., 1993; Gecz et al., 1999).

## Discussion

The modulation of excitatory pyramidal cells by GABAergic interneurons is determined by interneuron diversity, allowing for the complex computations performed by these neuronal circuits (Tremblay et al., 2016). Of the many interneuron subtypes, those that release GABA onto the soma and axon can powerfully influence and fine-tune neuronal activity (Miles et al., 1996). CCK positive cells target the soma and proximal dendrites of pyramidal cells in cortex and hippocampus, and rely on DG for targeting of α2/α3 containing GABA_A_Rs to postsynaptic sites on the hippocampal pyramidal cell soma (Früh et al., 2016). Interestingly, clustering of GABA_A_Rs at sites opposing CCK positive terminals appears to be independent of DG, as α2 containing GABA_A_Rs still cluster in DG cKO mice in the absence of CCK positive terminals (Früh et al., 2016). Consistent with this, gephyrin clustering was also unaffected in DG cKO, while CB was not assessed (Früh et al., 2016). It remains unclear how gephyrin might selectively stabilize specific subtypes of GABA_A_Rs at postsynaptic sites despite its ubiquitous presence, but leading hypotheses point to posttranslational modification (Ghosh et al., 2016), or subtleties in multi-protein complex arrangements (Saiepour et al., 2010). Somatic contacts from CCK positive basket cells were unaffected by a DG mutation that interferes with neurexin binding (Früh et al., 2016), encouraging the exploration of possible novel presynaptic partners in CCK positive terminals that are needed for transsynaptic signaling during synapse formation and maintenance at sites contacting the soma.

PV positive chandelier cells terminate onto the AIS which is enriched with GABA_A_Rs containing the α2 subunit, and the high affinity interaction between α2 and CB appears essential in this enrichment and in the maintenance of these synapses on cortical pyramidal cells (Hines et al., 2018). Despite evidence of a preferential interaction between α2 and CB, as well as a preferential role of this complex at AIS synapses onto cortical pyramidal cells, several points remain to be clarified. Although the interaction strength between α2 and gephyrin was comparatively weak, a prominent effect of α2 KO is a loss of gephyrin clustering at perisomatic synapses onto CA1 pyramidal cells (Panzanelli et al., 2011). The molecular mechanism regulating this loss of gephyrin remains unclear but may relate to an indirect interaction between α2 and gephyrin. Although the interaction between α2 and CB appears critical for AIS synapses in the cortex, α2 and CB are well known to be present at other synapse types. Conversely, α1 and α3 containing receptors can also be detected at AIS synapses, particularly in the hippocampus and amygdala (Gao and Heldt, 2016), thus analysis of the impact of the *Gabra2*-1 mutation on AIS synapses in other brain regions is needed.

Developmental RNA-seq focusing on the period of peak inhibitory synapse formation demonstrated that distinct types of interneurons rely on a largely unique complement of molecular programs for the specific subcellular contact sites that they establish (Favuzzi et al., 2019). During synaptogenesis, genes involved in targeting and matching PV positive interneuron axons to their postsynaptic targets include expression of *Lgi2* for basket cells, and *Fgf13* for chandelier cells (Favuzzi et al., 2019). Details of how the expressed proteins function at presynaptic terminals during synaptogenesis remain to be uncovered, including further illumination of specific interacting partners and effectors. In addition to the genes that were characterized in more detail, a number of others were identified to have relatively specific regulated expression patterns related to synapse formation (Favuzzi et al., 2019). Many of these were genes encoding adhesion proteins, as well as extracellular components such as proteins that make up the peri-neuronal net (Favuzzi et al., 2019). Investigation into some of the other candidates will allow for more detailed illumination of the steps involved in building each unique type of inhibitory contact on the soma and axon.

In general, studies have yet to replicate or contrast these mechanisms in regulating somatic and axon targeting inhibitory synapse formation, maintenance, and function in distinct brain circuits. As a point of comparison, the cerebellum has a more limited repertoire of cell types, and the cellular and molecular specialization of soma and axon targeting interneurons in the cerebellum is relatively well established (Somogyi and Hámori, 1976; Somogyi et al., 1983; Li et al., 1992; Ango et al., 2004). In the cerebellum, principal Purkinje cells receive GABAergic innervation from stellate and basket cells. Stellate cells target dendritic domains, while basket cells innervate the perisomatic region and ensheath the AIS (pinceau formation) of Purkinje cells. Guidance of the basket cell axon to the Purkinje AIS is mediated by Semaphorin 3A and its receptor neuropilin-1, which interacts directly with the adhesion molecule NF186 at the AIS target (Cioni et al., 2013; Telley et al., 2016). Maturation of the Purkinje AIS and pinceau formation relies upon neurofascin interaction with Ankyrin-G (Ango et al., 2004; Zonta et al., 2011; Buttermore et al., 2012). Somatic synapses on Purkinje cells are enriched with both α1 and α3 containing GABA_A_Rs (Fritschy et al., 2006). The maintenance of these synapses does not depend on α1 expression (Fritschy et al., 2006), and α1 expression on the Purkinje soma is maintained in CB knockout in the absence of gephyrin (Papadopoulos et al., 2007), leaving the mechanisms required to build the postsynaptic compartment of somatic synapses of Purkinje cells unclear. Also of interest, another intracellular FGF family member, FGF14, is localized to the AIS, and has been implicated in Purkinje neuron excitability by impacting voltage gated Na+ channel kinetics (Goldfarb et al., 2007; Xiao et al., 2013); thus distinct FGFs may play unique but complementary roles at the AIS. Additional studies should compare and contrast the contributions of this subclass of FGFs in formation and maintenance of axon targeting synapses across multiple circuits.

The function of inhibitory synapses on the soma and axon is perhaps best illustrated by the effects observed upon mutation (Table 1). Epilepsy is an interesting common thread among soma and axon targeting inhibitory synapse gene syndromes. Given the role of soma and axon targeting interneurons in coordinating principal cell activity, discoordination of neuronal activity patterns is a logical extension. Yet further studies are needed to understand the distinction between disrupting specific synapse subtypes and functional implications for circuit activity. Examination of animal models for these disorders focusing on abnormalities in the development and maintenance of specific inhibitory synapse subtypes will be helpful in confirming a selective contribution. Identification of specific synapse subtypes, along with key molecular players at these sites may allow the development of molecular and pharmacological interventions that more precisely modulate the development and maintenance of specific inhibitory synapse subtypes. Further knowledge of specific synapse subtypes in these disorders will ultimately aid with the refinement or development of novel therapeutic strategies.

## Author Contributions

AC, DH, and RH wrote the manuscript. RH conceived the work and prepared the figure.

## Conflict of Interest Statement

The authors declare that the research was conducted in the absence of any commercial or financial relationships that could be construed as a potential conflict of interest.

## References

[B1] Ali Rodriguez R. Joya C. Hines R. M. (2018). Common ribs of inhibitory synaptic dysfunction in the umbrella of neurodevelopmental disorders. *Front. Mol. Neurosci.* 11:132. 10.3389/fnmol.2018.00132 29740280 PMC5928253

[B2] Ango F. di Cristo G. Higashiyama H. Bennett V. Wu P. Huang Z. J. (2004). Ankyrin-based subcellular gradient of neurofascin, an immunoglobulin family protein, directs GABAergic innervation at purkinje axon initial segment. *Cell* 119 257–272. 10.1016/j.cell.2004.10.004 15479642

[B3] Barresi R. Campbell K. P. (2006). Dystroglycan: from biosynthesis to pathogenesis of human disease. *J. Cell Sci.* 119 199–207. 10.1242/jcs.02814 16410545

[B4] Baulac S. Huberfeld G. Gourfinkel-An I. Mitropoulou G. Beranger A. Prud’homme J. F. (2001). First genetic evidence of GABA(A) receptor dysfunction in epilepsy: a mutation in the gamma2-subunit gene. *Nat. Genet.* 28 46–48. 10.1038/88254 11326274

[B5] Brancaccio A. (2005). α-Dystroglycan, the usual suspect? *Neuromuscul. Disord.* 15 825–828. 10.1016/j.nmd.2005.08.003 16289897

[B6] Brünig I. Suter A. Knuesel I. Lüscher B. Fritschy J.-M. (2002). GABAergic terminals are required for postsynaptic clustering of dystrophin but not of GABAA receptors and gephyrin. *J. Neurosci.* 22 4805–4813. 10.1523/JNEUROSCI.22-12-04805.2002 12077177 PMC6757720

[B7] Buttermore E. D. Piochon C. Wallace M. L. Philpot B. D. Hansel C. Bhat M. A. (2012). Pinceau organization in the cerebellum requires distinct functions of neurofascin in Purkinje and basket neurons during postnatal development. *J. Neurosci. Off. J. Soc. Neurosci.* 32 4724–4742. 10.1523/JNEUROSCI.5602-11.2012 22492029 PMC3337041

[B8] Buzsáki G. (1996). The hippocampo-neocortical dialogue. *Cereb. Cortex* 6 81–92. 10.1093/cercor/6.2.81 8670641

[B9] Cajal S. (1893). Estructura del asta de Ammon. *Ann. Soc Esp Hist Nat. Madr.* 22 53–114.

[B10] Cioni J.-M. Telley L. Saywell V. Cadilhac C. Jourdan C. Huber A. B. (2013). SEMA3A signaling controls layer-specific interneuron branching in the cerebellum. *Curr. Biol.* 23 850–861. 10.1016/j.cub.2013.04.007 23602477

[B11] Daoud F. Angeard N. Demerre B. Martie I. Benyaou R. Leturcq F. (2009). Analysis of Dp71 contribution in the severity of mental retardation through comparison of Duchenne and Becker patients differing by mutation consequences on Dp71 expression. *Hum. Mol. Genet.* 18 3779–3794. 10.1093/hmg/ddp320 19602481

[B12] DeFelipe J. Garcia Sola R. Marco P. del Río M. R. Pulido P. Ramón y Cajal S. (1993). Selective changes in the microorganization of the human epileptogenic neocortex revealed by parvalbumin immunoreactivity. *Cereb. Cortex* 3 39–48. 10.1093/cercor/3.1.39 7679938

[B13] Defelipe J. Hendry S. H. C. Jones E. G. Schmechel D. (1985). Variability in the terminations of GABAergic chandelier cell axons on initial segments of pyramidal cell axons in the monkey sensory-motor cortex. *J. Comp. Neurol.* 231 364–384. 10.1002/cne.902310307 2981907

[B14] Desguerre I. Christov C. Mayer M. Zeller R. Becane H.-M. Bastuji-Garin S. (2009). Clinical heterogeneity of duchenne muscular dystrophy (DMD): definition of sub-phenotypes and predictive criteria by long-term follow-up. *PLoS One* 4:e4347. 10.1371/journal.pone.0004347 19194511 PMC2633042

[B15] Favuzzi E. Deogracias R. Marques-Smith A. Maeso P. Jezequel J. Exposito-Alonso D. (2019). Distinct molecular programs regulate synapse specificity in cortical inhibitory circuits. *Science* 363 413–417. 10.1126/science.aau8977 30679375

[B16] Freund T. F. Buzsáki G. (1996). Interneurons of the hippocampus. *Hippocampus* 6 347–470. 10.1002/(sici)1098-1063(1996)6:4<347::aid-hipo1>3.0.co;2-i 8915675

[B17] Freund T. F. Katona I. (2007). Perisomatic inhibition. *Neuron* 56 33–42. 10.1016/j.neuron.2007.09.012 17920013

[B18] Fritschy J.-M. Panzanelli P. Kralic J. E. Vogt K. E. Sassoè-Pognetto M. (2006). Differential dependence of axo-dendritic and axo-somatic GABAergic synapses on GABAA receptors containing the alpha1 subunit in purkinje cells. *J. Neurosci. Off. J. Soc. Neurosci.* 26 3245–3255. 10.1523/JNEUROSCI.5118-05.2006 16554475 PMC6674111

[B19] Früh S. Romanos J. Panzanelli P. Bürgisser D. Tyagarajan S. K. Campbell K. P. (2016). Neuronal dystroglycan is necessary for formation and maintenance of functional CCK-positive basket cell terminals on pyramidal cells. *J. Neurosci. Off. J. Soc. Neurosci.* 36 10296–10313. 10.1523/JNEUROSCI.1823-16.2016 27707967 PMC6705590

[B20] Fukata Y. Lovero K. L. Iwanaga T. Watanabe A. Yokoi N. Tabuchi K. (2010). Disruption of LGI1–linked synaptic complex causes abnormal synaptic transmission and epilepsy. *Proc. Natl. Acad. Sci. U.S.A.* 107 3799–3804. 10.1073/pnas.0914537107 20133599 PMC2840530

[B21] Gao Y. Heldt S. A. (2016). Enrichment of GABAA receptor α-subunits on the axonal initial segment shows regional differences. *Front. Cell. Neurosci.* 10:39 10.3389/fncel.2016.00039 PMC4771769 26973458

[B22] Gecz J. Baker E. Donnelly A. Ming J. E. McDonald-McGinn D. M. Spinner N. B. (1999). Fibroblast growth factor homologous factor 2 (FHF2): gene structure, expression and mapping to the Börjeson-Forssman-Lehmann syndrome region in Xq26 delineated by a duplication breakpoint in a BFLS-like patient. *Hum. Genet.* 104 56–63. 10.1007/s004390050910 10071193

[B23] Ghosh H. Auguadri L. Battaglia S. Simone Thirouin Z. Zemoura K. Messner S. (2016). Several posttranslational modifications act in concert to regulate gephyrin scaffolding and GABAergic transmission. *Nat. Commun.* 7:13365. 10.1038/ncomms13365 27819299 PMC5103071

[B24] Goldfarb M. Schoorlemmer J. Williams A. Diwakar S. Wang Q. Huang X. (2007). Fibroblast growth factor homologous factors control neuronal excitability through modulation of voltage-gated sodium channels. *Neuron* 55 449–463. 10.1016/j.neuron.2007.07.006 17678857 PMC2974323

[B25] Guillemot F. Zimmer C. (2011). From cradle to grave: the multiple roles of fibroblast growth factors in neural development. *Neuron* 71 574–588. 10.1016/j.neuron.2011.08.002 21867876

[B26] Hines R. M. Hines D. J. Houston C. M. Mukherjee J. Haydon P. G. Tretter V. (2013). Disrupting the clustering of GABAA receptor α2 subunits in the frontal cortex leads to reduced γ-power and cognitive deficits. *Proc. Natl. Acad. Sci. U.S.A.* 110 16628–16633. 10.1073/pnas.1308706110 24043839 PMC3799382

[B27] Hines R. M. Maric H. M. Hines D. J. Modgil A. Panzanelli P. Nakamura Y. (2018). Developmental seizures and mortality result from reducing GABAA receptor α2-subunit interaction with collybistin. *Nat. Commun.* 9:3130. 10.1038/s41467-018-05481-1 30087324 PMC6081406

[B28] Hu H. Gan J. Jonas P. (2014). Fast-spiking, parvalbumin+ GABAergic interneurons: from cellular design to microcircuit function. *Science* 345:1255263. 10.1126/science.1255263 25082707

[B29] Jacob T. C. Moss S. J. Jurd R. (2008). GABAA receptor trafficking and its role in the dynamic modulation of neuronal inhibition. *Nat. Rev. Neurosci.* 9 331–343. 10.1038/nrn2370 18382465 PMC2709246

[B30] Kalachikov S. Evgrafov O. Ross B. Winawer M. Barker-Cummings C. Boneschi F. M. (2002). Mutations in *LGI1* cause autosomal-dominant partial epilepsy with auditory features. *Nat. Genet.* 30 335–341. 10.1038/ng832 11810107 PMC2606053

[B31] Kalscheuer V. M. Musante L. Fang C. Hoffmann K. Fuchs C. Carta E. (2009). A balanced chromosomal translocation disrupting ARHGEF9 is associated with epilepsy, anxiety, aggression, and mental retardation. *Hum. Mutat.* 30 61–68. 10.1002/humu.20814 18615734 PMC3621145

[B32] Katona I. Sperlágh B. Sík A. Käfalvi A. Vizi E. S. Mackie K. (1999). Presynaptically located CB1 cannabinoid receptors regulate GABA release from axon terminals of specific hippocampal interneurons. *J. Neurosci. Off. J. Soc. Neurosci.* 19 4544–4558. 10.1523/jneurosci.19-11-04544.1999 10341254 PMC6782612

[B33] Kegel L. Aunin E. Meijer D. Bermingham J. R. (2013). LGI proteins in the nervous system. *ASN Neuro* 5:AN20120095. 10.1042/AN20120095 23713523 PMC3691968

[B34] Kinton L. Johnson M. R. Smith S. J. M. Farrell F. Stevens J. Rance J. B. (2002). Partial epilepsy with pericentral spikes: a new familial epilepsy syndrome with evidence for linkage to chromosome 4p15. *Ann. Neurol.* 51 740–749. 10.1002/ana.10221 12112080

[B35] Klausberger T. Somogyi P. (2008). Neuronal diversity and temporal dynamics: the unity of hippocampal circuit operations. *Science* 321 53–57. 10.1126/science.1149381 18599766 PMC4487503

[B36] Knuesel I. Mastrocola M. Zuellig R. A. Bornhauser B. Schaub M. C. Fritschy J. M. (1999). Short communication: altered synaptic clustering of GABAA receptors in mice lacking dystrophin (mdx mice). *Eur. J. Neurosci.* 11 4457–4462. 10.1046/j.1460-9568.1999.00887.x 10594673

[B37] Kubota Y. Karube F. Nomura M. Kawaguchi Y. (2016). The diversity of cortical inhibitory synapses. *Front. Neural Circ.* 10:27. 10.3389/fncir.2016.00027 27199670 PMC4842771

[B38] Lee S.-H. Soltesz I. (2011). Requirement for CB1 but not GABAB receptors in the cholecystokinin mediated inhibition of GABA release from cholecystokinin expressing basket cells. *J. Physiol.* 589 891–902. 10.1113/jphysiol.2010.198499 21173082 PMC3060368

[B39] Lévi S. Grady R. M. Henry M. D. Campbell K. P. Sanes J. R. Craig A. M. (2002). Dystroglycan is selectively associated with inhibitory GABAergic synapses but is dispensable for their differentiation. *J. Neurosci.* 22 4274–4285. 10.1523/JNEUROSCI.22-11-04274.2002 12040032 PMC6758826

[B40] Lewis D. A. Cho R. Y. Carter C. S. Eklund K. Forster S. Kelly M. A. (2008). Subunit-selective modulation of GABA type a receptor neurotransmission and cognition in schizophrenia. *Am. J. Psychiatry* 165 1585–1593. 10.1176/appi.ajp.2008.08030395 18923067 PMC2876339

[B41] Lewis D. A. Curley A. A. Glausier J. R. Volk D. W. (2012). Cortical parvalbumin interneurons and cognitive dysfunction in schizophrenia. *Trends Neurosci.* 35 57–67. 10.1016/j.tins.2011.10.004 22154068 PMC3253230

[B42] Li X. G. Somogyi P. Tepper J. M. Buzsáki G. (1992). Axonal and dendritic arborization of an intracellularly labeled chandelier cell in the CA1 region of rat hippocampus. *Exp. Brain Res.* 90 519–525. 1385200 10.1007/BF00230934

[B43] Limviphuvadh V. Chua L. L. Rahim R. A. A. B. Eisenhaber F. Maurer-Stroh S. Adhikari S. (2010). Similarity of molecular phenotype between known epilepsy gene LGI1 and disease candidate gene LGI2. *BMC Biochem.* 11:39. 10.1186/1471-2091-11-39 20863412 PMC2949613

[B44] Lorente and De N ó R. (1934). Studies on the structure of the cerebral cortex. II. Continuation of the study of the ammonic system. *J. Für Psychol. Neurol.* 46 113–177.

[B45] Malmgren H. Sundvall M. Dahl N. Gustavson K. H. Annerén G. Wadelius C. (1993). Linkage mapping of a severe X-linked mental retardation syndrome. *Am. J. Hum. Genet.* 52 1046–1052. 8503440 PMC1682275

[B46] McNally E. M. Pytel P. (2007). Muscle diseases: the muscular dystrophies. *Annu. Rev. Pathol. Mech. Dis.* 2 87–109. 10.1146/annurev.pathol.2.010506.091936 18039094

[B47] Miles R. Tóth K. Gulyás A. I. Hájos N. Freund T. F. (1996). Differences between somatic and dendritic inhibition in the hippocampus. *Neuron* 16 815–823. 10.1016/s0896-6273(00)80101-4 8607999

[B48] Moore S. A. Saito F. Chen J. Michele D. E. Henry M. D. Messing A. (2002). Deletion of brain dystroglycan recapitulates aspects of congenital muscular dystrophy. *Nature* 418 422–425. 10.1038/nature00838 12140559

[B49] Morales M. Bloom F. E. (1997). The 5-HT3 receptor is present in different subpopulations of GABAergic neurons in the rat telencephalon. *J. Neurosci. Off. J. Soc. Neurosci.* 17 3157–3167. 10.1523/jneurosci.17-09-03157.1997 9096150 PMC6573651

[B50] Nagode D. A. Tang A.-H. Yang K. Alger B. E. (2014). Optogenetic identification of an intrinsic cholinergically driven inhibitory oscillator sensitive to cannabinoids and opioids in hippocampal CA1. *J. Physiol.* 592 103–123. 10.1113/jphysiol.2013.257428 24190932 PMC3903354

[B51] Nusser Z. Sieghart W. Benke D. Fritschy J. M. Somogyi P. (1996). Differential synaptic localization of two major gamma-aminobutyric acid type A receptor alpha subunits on hippocampal pyramidal cells. *Proc. Natl. Acad. Sci. U.S.A.* 93 11939–11944. 10.1073/pnas.93.21.11939 8876241 PMC38162

[B52] Nyíri G. Freund T. F. Somogyi P. (2001). Input-dependent synaptic targeting of alpha(2)-subunit-containing GABA(A) receptors in synapses of hippocampal pyramidal cells of the rat. *Eur. J. Neurosci.* 13 428–442. 10.1046/j.1460-9568.2001.01407.x 11168550

[B53] Pablo J. L. Wang C. Presby M. M. Pitt G. S. (2016). Polarized localization of voltage-gated Na+ channels is regulated by concerted FGF13 and FGF14 action. *Proc. Natl. Acad. Sci. U.S.A.* 113 E2665–E2674. 10.1073/pnas.1521194113 27044086 PMC4868475

[B54] Panzanelli P. Gunn B. G. Schlatter M. C. Benke D. Tyagarajan S. K. Scheiffele P. (2011). Distinct mechanisms regulate GABAA receptor and gephyrin clustering at perisomatic and axo-axonic synapses on CA1 pyramidal cells. *J. Physiol.* 589 4959–4980. 10.1113/jphysiol.2011.216028 21825022 PMC3224886

[B55] Papadopoulos T. Korte M. Eulenburg V. Kubota H. Retiounskaia M. Harvey R. J. (2007). Impaired GABAergic transmission and altered hippocampal synaptic plasticity in collybistin-deficient mice. *EMBO J.* 26 3888–3899. 10.1038/sj.emboj.7601819 17690689 PMC1994120

[B56] Parra P. Gulyás A. I. Miles R. (1998). How many subtypes of inhibitory cells in the hippocampus? *Neuron* 20 983–993. 10.1016/s0896-6273(00)80479-1 9620702

[B57] Petilla Interneuron Nomenclature Group Ascoli G. A. Alonso-Nanclares L. Anderson S. A. Barrionuevo G. Benavides-Piccione R. (2008). Petilla terminology: nomenclature of features of GABAergic interneurons of the cerebral cortex. *Nat. Rev. Neurosci.* 9 557–568. 10.1038/nrn2402 18568015 PMC2868386

[B58] Puranam R. S. He X. P. Yao L. Le T. Jang W. Rehder C. W. (2015). Disruption of Fgf13 causes synaptic excitatory-inhibitory imbalance and genetic epilepsy and febrile seizures plus. *J. Neurosci.* 35 8866–8881. 10.1523/JNEUROSCI.3470-14.2015 26063919 PMC4461691

[B59] Rubenstein J. L. R. Merzenich M. M. (2003). Model of autism: increased ratio of excitation/inhibition in key neural systems. *Genes Brain Behav.* 2 255–267. 10.1034/j.1601-183X.2003.00037.x 14606691 PMC6748642

[B60] Saiepour L. Fuchs C. Patrizi A. Sassoè-Pognetto M. Harvey R. J. Harvey K. (2010). Complex role of collybistin and gephyrin in GABAA receptor clustering. *J. Biol. Chem.* 285 29623–29631. 10.1074/jbc.M110.121368 20622020 PMC2937993

[B61] Senechal K. R. Thaller C. Noebels J. L. (2005). ADPEAF mutations reduce levels of secreted LGI1, a putative tumor suppressor protein linked to epilepsy. *Hum. Mol. Genet.* 14 1613–1620. 10.1093/hmg/ddi169 15857855

[B62] Seppälä E. H. Jokinen T. S. Fukata M. Fukata Y. Webster M. T. Karlsson E. K. (2011). LGI2 truncation causes a remitting focal epilepsy in dogs. *PLoS Genet.* 7:e1002194. 10.1371/journal.pgen.1002194 21829378 PMC3145619

[B63] Shimojima K. Sugawara M. Shichiji M. Mukaida S. Takayama R. Imai K. (2011). Loss-of-function mutation of collybistin is responsible for X-linked mental retardation associated with epilepsy. *J. Hum. Genet.* 56 561–565. 10.1038/jhg.2011.58 21633362

[B64] Somogyi P. Hámori J. (1976). A quantitative electron microscopic study of the purkinje cell axon initial segment. *Neuroscience* 1 361–365. 1004711 10.1016/0306-4522(76)90127-5

[B65] Somogyi P. Nunzi M. G. Gorio A. Smith A. D. (1983). A new type of specific interneuron in the monkey hippocampus forming synapses exclusively with the axon initial segments of pyramidal cells. *Brain Res.* 259 137–142. 10.1016/0006-8993(83)91076-4 6824927

[B66] Striano P. Zara F. (2017). ARHGEF9 mutations cause a specific recognizable X-linked intellectual disability syndrome. *Neurol. Genet.* 3:e159. 10.1212/NXG.0000000000000159 28589177 PMC5446781

[B67] Sugita S. Saito F. Tang J. Satz J. Campbell K. Südhof T. C. (2001). A stoichiometric complex of neurexins and dystroglycan in brain. *J. Cell Biol.* 154 435–446. 10.1083/jcb.200105003 11470830 PMC2150755

[B68] Tasic B. Menon V. Nguyen T. N. Kim T. K. Jarsky T. Yao Z. (2016). Adult mouse cortical cell taxonomy revealed by single cell transcriptomics. *Nat. Neurosci.* 19 335–346. 10.1038/nn.4216 26727548 PMC4985242

[B69] Telley L. Cadilhac C. Cioni J.-M. Saywell V. Jahannault-Talignani C. Huettl R. E. (2016). Dual function of NRP1 in axon guidance and subcellular target recognition in cerebellum. *Neuron* 91 1276–1291. 10.1016/j.neuron.2016.08.015 27618676

[B70] The International League Against Epilepsy Consortium on Complex Epilepsies (2018). Genome-wide mega-analysis identifies 16 loci and highlights diverse biological mechanisms in the common epilepsies. *Nat. Commun.* 9:5269. 10.1038/s41467-018-07524-z 30531953 PMC6288131

[B71] Tremblay R. Lee S. Rudy B. (2016). GABAergic interneurons in the neocortex: from cellular properties to circuits. *Neuron* 91 260–292. 10.1016/j.neuron.2016.06.033 27477017 PMC4980915

[B72] Vaillend C. Perronnet C. Ros C. Gruszczynski C. Goyenvalle A. Laroche S. (2010). Rescue of a dystrophin-like protein by exon skipping in vivo restores GABAA-receptor clustering in the hippocampus of the mdx mouse. *Mol. Ther. J. Am. Soc. Gene Ther.* 18 1683–1688. 10.1038/mt.2010.134 20588257 PMC2956917

[B73] Wallace R. H. Marini C. Petrou S. Harkin L. A. Bowser D. N. Panchal R. G. (2001). Mutant GABA(A) receptor gamma2-subunit in childhood absence epilepsy and febrile seizures. *Nat. Genet.* 28 49–52. 10.1038/88259 11326275

[B74] Wang J.-Y. Zhou P. Wang J. Tang B. Su T. Liu X.-R. (2018). ARHGEF9 mutations in epileptic encephalopathy/intellectual disability: toward understanding the mechanism underlying phenotypic variation. *Neurogenetics* 19 9–16. 10.1007/s10048-017-0528-2 29130122

[B75] Weber E. T. Andrade R. (2010). Htr2a gene and 5-HT2A receptor expression in the cerebral cortex studied using genetically modified mice. *Front. Neurosci.* 4:36. 10.3389/fnins.2010.00036 20802802 PMC2928707

[B76] Whissell P. D. Cajanding J. D. Fogel N. Kim J. C. (2015). Comparative density of CCK- and PV-GABA cells within the cortex and hippocampus. *Front. Neuroanat.* 9:124. 10.3389/fnana.2015.00124 26441554 PMC4585045

[B77] Wu Q.-F. Yang L. Li S. Wang Q. Yuan X.-B. Gao X. (2012). Fibroblast growth factor 13 is a microtubule-stabilizing protein regulating neuronal polarization and migration. *Cell* 149 1549–1564. 10.1016/j.cell.2012.04.046 22726441

[B78] Xiao M. Bosch M. K. Nerbonne J. M. Ornitz D. M. (2013). FGF14 localization and organization of the axon initial segment. *Mol. Cell. Neurosci.* 56 393–403. 10.1016/j.mcn.2013.07.008 23891806 PMC3791165

[B79] Zonta B. Desmazieres A. Rinaldi A. Tait S. Sherman D. L. Nolan M. F. (2011). A critical role for neurofascin in regulating action potential initiation through maintenance of the axon initial segment. *Neuron* 69 945–956. 10.1016/j.neuron.2011.02.021 21382554 PMC3057015

